# Delivery of miRNA-Targeted Oligonucleotides in the Rat Striatum by Magnetofection with Neuromag^®^

**DOI:** 10.3390/molecules23071825

**Published:** 2018-07-23

**Authors:** Simoneide Souza Titze de Almeida, Camila Hillesheim Horst, Cristina Soto-Sánchez, Eduardo Fernandez, Ricardo Titze de Almeida

**Affiliations:** 1 Technology for Gene Therapy Laboratory, Central Institute of Sciences, FAV, University of Brasilia, Brasília 70910-900, Brazil; simoneide.silva@gmail.com (S.S.T.d.A.); horstcamila@gmail.com (C.H.H.); 2 Neuroprosthetics and Visual Rehabilitation Research Unit, Bioengineering Institute, Miguel Hernández University, 03202 Alicante, Spain; csoto@goumh.umh.es (C.S.-S.); e.fernandez@umh.es (E.F.); 3 Biomedical Research Networking Center in Bioengineering, Biomaterials and Nanomedicine—CIBER-BBN, 28029 Madrid, Spain; 4 Biotechnology Department, University of Alicante, 03080 Alicante, Spain

**Keywords:** miRNA, nanoparticles, Neuromag^®^, delivery

## Abstract

MicroRNAs (miRNAs) regulate gene expression at posttranscriptional level by triggering RNA interference. In such a sense, aberrant expressions of miRNAs play critical roles in the pathogenesis of many disorders, including Parkinson’s disease (PD). Controlling the level of specific miRNAs in the brain is thus a promising therapeutic strategy for neuroprotection. A fundamental need for miRNA regulation (either replacing or inhibition) is a carrier capable of delivering oligonucleotides into brain cells. This study aimed to examine a polymeric magnetic particle, Neuromag^®^, for delivery of synthetic miRNA inhibitors in the rat central nervous system. We injected the miRNA inhibitor complexed with Neuromag^®^ into the lateral ventricles next to the striatum, by stereotaxic surgery. Neuromag efficiently delivered oligonucleotides in the striatum and septum areas, as shown by microscopy imaging of fluorescein isothiocyanate (FITC)-labeled oligos in astrocytes and neurons. Transfected oligos showed efficacy concerning miRNA inhibition. Neuromag^®^-structured miR-134 antimiR (0.36 nmol) caused a significant 0.35 fold decrease of striatal miR-134, as revealed by real-time quantitative polymerase chain reaction (RT-qPCR). In conclusion, the polymeric magnetic particle Neuromag^®^ efficiently delivered functional miRNA inhibitors in brain regions surrounding lateral ventricles, particularly the striatum. This delivery system holds potential as a promising miRNA-based disease-modifying drug and merits further pre-clinical studies using animal models of PD.

## 1. Introduction

MicroRNAs (miRNAs) are endogenous short noncoding RNAs that cause post-transcriptional gene silencing in healthy cells. Knocking down first requires the binding of miRNA guide strands, complexed with silencing proteins, to complementary sequences in 3′-untranslated region (3′-UTR) of the target mRNAs. This silencing complex will ultimately cause translational repression or mRNA degradation [1]. RNAi is thus a crucial mechanism of gene regulation. Conversely, aberrant expressions of miRNAs play critical roles in the neuropathology of brain diseases [2]. Therefore, exogenous synthetic oligonucleotides have been used to modulate the content of specific miRNAs. Antisense single-stranded oligos (AntimiRs) are capable of annealing to the guide strand of mature miRNAs, forming hetero-duplexes devoided of RNAi activity [1,3]. 

Regarding brain diseases, AntimiRs were first used to suppress the overexpression of miR-134 in brain areas related to cell death and epileptogenesis [4]. A seminal work of Henshall′s group reported the efficacy of AntimiR-134 injected by the intracerebroventricular (i.c.v.) route in reducing the loss of hippocampal cells and the number of seizures in the model of epilepsy triggered by intra-amygdalar injection of kainic acid [5]. The neuroprotective effects of brain-injected miR-134-targeted AntimiRs were corroborated in other models of temporal lobe epilepsy, including pilocarpine, pentylenetetrazol, and perforant pathway stimulation [6,7]. Indeed, our group found that AntimiRs also attenuated the injury of dopaminergic SH-SY5Y cells triggered by rotenone, a cellular model of Parkinson’s disease (PD) [8]. Therefore, an i.c.v. injection of AntimiRs might protect neurons in the rat striatum, a region critically involved in PD neuropathology. 

Although many synthetic oligonucleotides have shown clinical efficacy in a broad range of pathologies, transfection of neuronal cells for treating brain diseases remains a prominent challenge [9]. The blood-brain barrier and the complex cellular organization in the brain make the central nervous system (CNS) a pharmacological biophase relatively difficult to reach. In such sense, viral vectors remain predominantly used in clinical trials due to their higher transfecting efficiency [10]. However, these platforms hold disadvantages such as induction of inflammatory responses and potential mutagenic effects in the host [11,12]. Non-viral vectors, in contrast, present lower immunogenicity and higher nucleic acid packing capacity [13]. Additionally, non-viral formulations can be produced on a large scale with high reproducibility and acceptable costs, with relative stability for storage [14]. In this context, the magnetofection technology has employed advanced functional nanocarriers with an increased transfection rate in neurons. The principle of this technology consists of a magnetic nanoparticle set up with magnetic fields that will drive gene delivery [15]. These particles contain biocompatible materials with a magnetic core and are capable of carrying nucleic acid molecules, such as DNA plasmids, oligonucleotides, siRNAs, and miRNAs [16,17]. The principal advantage of such a delivery system is that the endocytotic uptake mechanism—the transfection—is markedly enhanced by magnetic fields, thus the nomination “magnetofection” [15]. 

**Scheme 1:**
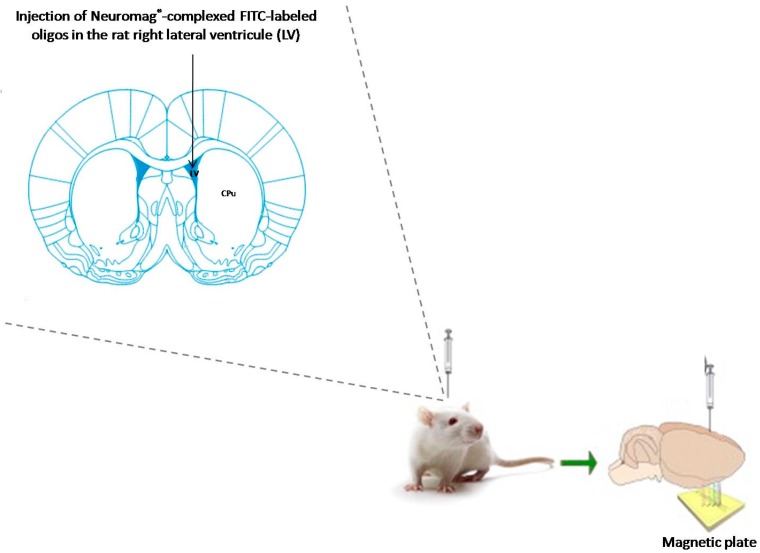
Schematic view of magnetofection for delivering oligonucleotides in the rat brain, based on previous work [18]. Neuromag^®^-complexed oligos were injected by stereotaxic surgery in the right lateral ventricle, in coordinates according to the Paxinos atlas. A magnetic plate placed underneath the head enhances transfection of magnetic particles into the neuron and glial cells. Detailed information in material and methods section (item 4.1). CPu—caudate putamen; LV—lateral ventricle.

Our group described for the first time the capacity of NeuroMag^®^—the magnetic particle employed in the present study, to deliver nucleic acids in vivo into rat brain cells [18]. This polymeric carrier took advantage of magnetic fields and delivered plasmids selectively into neuronal cells of the rat visual cortex. No previous work, however, has yet tested whether NeuroMag^®^ displays an ability to transfect cells with functional short antisense oligonucleotides for miRNA inhibition. In the present study, we examined whether NeuroMag^®^-complexed miR-134 inhibitors injected by the i.c.v. route would efficiently knock-down miR-134 in the rat striatum, a brain region directly involved in Parkinson′s disease neuropathology (Scheme 1). 

## 2. Results

### 2.1. Magnetoparticle Characterization

The Neuromag^®^ magnetic nanoparticles and the magnetoplexes Neuromag^®^-complexed oligos were determined in terms of size, polydispersity index (PDI) and zeta potential. The pure magnetic nanoparticles’ average diameter was 188.84 nm with a polydispersity index of 0.25 and a superficial positive charge of +52.04 mV. After conjugation with the anti-miRNA to form magnetoplexes, the average diameter increased to 205.55 nm with a polydispersity index of 0.29 and the superficial charge decreased to −34.39 mV.

### 2.2. Transfection of Fluorescein Isothiocyanate (FITC)-Labeled Oligos in the Rat Striatum Cells by Using Neuromag^®^ and Magnetic Fields

**Figure 1:**
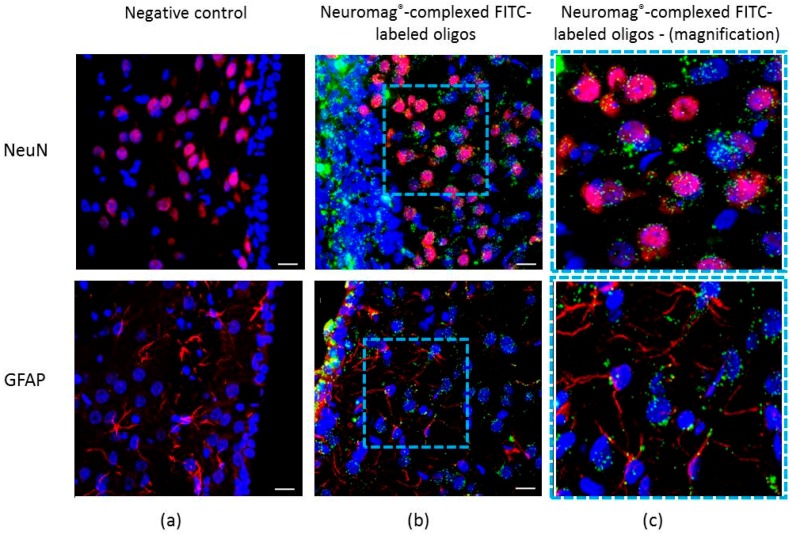
Magnetofection of striatal cells by fluorescein isothiocyanate (FITC)-labeled oligonucleotides complexed with Neuromag^®^. Striatum region transfected with oligos structured in Neuromag^®^ for delivery into the neuron and glial cells. (**a**) Left rat striatum without intracerebroventricular (i.c.v.) injection (negative control); (**b**) NeuN-positive neuron cells transfected with green fluorescent FITC-labeled oligos; (**c**) Delineated region of (**b**) in high magnification revealing the green-labeled oligonucleotide inside neurons and glial cells (upper and bottom images, respectively). Immunostaining of NeuN and GFAP in red; FITC-labeled oligos in green; cell nucleus in blue, stained by the Hoescht 33342 reagent. Scale = 15 µm. *n* = 3 per group.

This work then examined whether the intracerebroventricular (i.c.v.) route of administration would allow Neuromag^®^-complexed oligonucleotides to reach the striatum, a brain area strikingly relevant for PD. For that, we employed fluorescent fluorescein isothiocyanate (FITC)-labeled oligos i.c.v. injected and carried out microscope imaging. Magnetofection with Neuromag^®^ enhanced by magnetic plates was effective in driving the oligos across the ependymal cell layer adjacent to the right striatum, and for the subsequent delivery in striatal cells (Figure 1b). Green fluorescent FITC-labeled oligos were next to the cell nucleus of striatal neurons stained by NeuN, and also in GFAP-positive glial cells (Figure 1c, upper and bottom, respectively). As shown in Figure 1a, the contralateral noninjected hemisphere showed no FITC-oligonucleotide green fluorescence. This work proceeded to additional brain injections into the rat visual cortex, for comparison with the first in vivo testing of Neuromag^®^ injected in that region [18]. We also found positive fluorescence in apical dendrites and somas of the pyramidal cells (Figure S1). 

### 2.3. Silencing of miR-134 by the Neuromag^®^ -Complexed AntimiR-134

**Figure 2:**
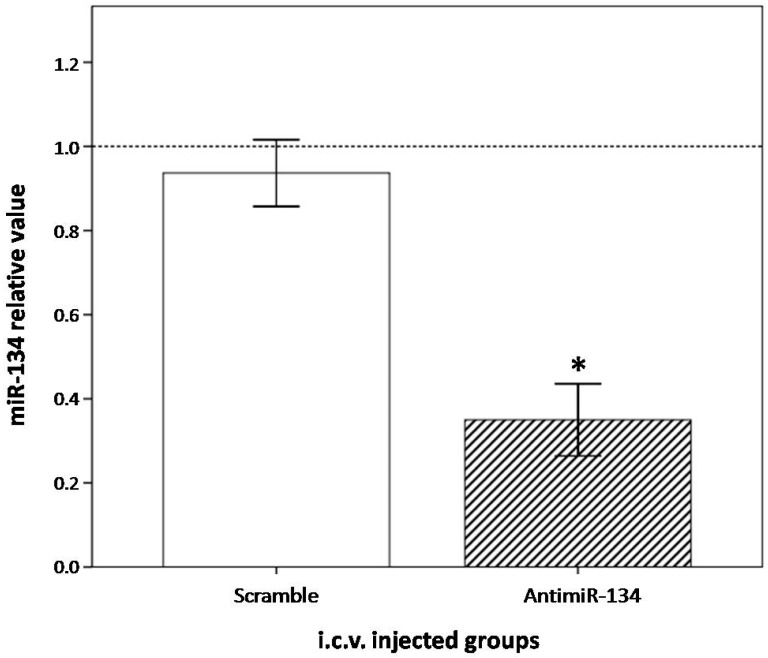
Knocking down on striatal miR-134 by Neuromag-complexed AntimiR-134 oligos. The content of miR-134 in the striatum determined by real-time quantitative polymerase chain reaction (RT-qPCR), at seven days after i.c.v. injections. miR-134 results in groups injected with AntimiR-134 or the negative control Scramble first normalized with RNU6B (∆Ct), and them compared to those obtained in control animals (∆∆Ct), arbitrarily assigned as 1.0 (100% expression). Bars represent mean ± SEM regarding miRNA relative value. * *P* < 0.05. *n* = 5 per group.

The imaging of FITC-labeled oligos described above (Figure 1) revealed a clear biodistribution from the injection site in lateral ventricle to surrounding tissues including striatum, an area critically involved in PD dopaminergic degeneration. Thus we examined whether an i.c.v. injection of AntimiR-134 + Neuromag^®^ would produce the desired effect, i.e., the knock-down on striatal miR-134. For that, we quantified by RT-qPCR the level of miR-134 in rat striatal tissues manually dissected at seven days after i.c.v. injection. AntimiR-134-injected animals presented a marked reduction of miR-134 in the striatum, as shown in Figure 2. Injecting 0.36 nmol of AntimiR-134 complexed with Neuromag^®^ caused a 0.35 fold decrease of this miRNA compared to controls, revealing an efficient and sustained knockdown (*P* < 0.05). Furthermore, injections of the nonsense scrambled miRNA sequences caused a small and not significant effect on miR-134 content. That means the miR-134 knockdown triggered by AntimiR-134 was sequence-dependent and target specific, which is crucial for achieving a further miRNA-based neuroprotective therapy.

## 3. Discussion

RNA interference (RNAi) is undoubtedly the most significant scientific discovery of the last two decades and is being exploited in many drugs for clinical testing [9]. The first one to complete the phase III will probably reach the pharmaceutical market this year, i.e., two decades after the seminal article of Fire and Melo awarded by Nobel Prize in Physiology or Medicine 2006 “for their discovery of RNA interference—gene silencing by double-stranded RNA” (https://www.nobelprize.org/nobel_prizes/medicine/laureates/2006/#) [19,20,21]. Following this outstanding work, many initial reports about RNAi focused fundamentally on suppressing genes of interest by using small-interfering RNAs (siRNAs) [22]. Our group has successfully silenced target genes by using different knocking-down strategies (i.e., synthetic oligos or short-hairpin expression vectors), working with both in vitro and in vivo assays, in neurological and non-neurological models [23,24,25,26,27]. More recently, however, a new class of RNAi molecules entered the scenario for playing an even more notorious role in science and technology: the microRNAs (miRNAs) [28,29]. miRNAs regulate mRNA content at a post-transcriptional level for aiding many vital functions in different biological systems, including embryonic development, cell maintenance, chemical signaling, and apoptosis, as revised elsewhere [3,30]. On the other hand, miRNAs aberrantly expressed contribute to underlying mechanisms of neurodegenerative diseases [31,32,33]. Controlling the content of pathological miRNAs by synthetic oligonucleotides (inhibitors or mimics) is thus a promising therapeutic strategy [4,34]. An illustrative example is miR-134, a microRNA implicated in health-disease processes including excitatory neurotransmission, neuritogenesis, spinal growth, and neuroplasticity [35,36,37,38,39]. Overexpression of miR-134 occurs in areas of brain degeneration related to epileptogenesis, and the silencing of this miRNA by i.c.v. injection of antisense oligos prevented both the loss of brain cells and the severity of the neurological process [5,6]. 

Since the early introduction of small-interfering molecules for RNAi-mediated gene silencing, two major challenges came about: the first was to provide a sustained effect on the targeted genes—which was achieved by chemical changes in RNA nucleotides [9,40]; the second was delivery [41,42]. The particle initially used was lipofectamine, a true liposome i.e., a lipid-vesicle capable of carrying nucleic acids into its inner aqueous core [43]. Our group has employed lipofectamine for delivering short-hairpin RNAi expression vectors and synthetic small-interfering RNAs (siRNAs) into astrocytic brain tumor cells in culture [24,25,26]. This carrier was also helpful for delivering siRNAs, as reported in dopaminergic neurons in vitro [27] and in a model of disk degeneration [23]. Lipid carriers evolved drastically, and many new features have improved particle stability, lysosomal scape, and even site-directed delivery particularly helpful for systemically-administered siRNAs now in advanced phases of clinical testing [9]. Non-lipid carriers also became available, and the poly(lactic-co-glycolic acid)—PLGA biodegradable polymeric particle is a representative example [44]. We reported that PLGA provides sustained delivery of drugs in vivo [45]. This carrier also enabled a continuous delivery of siRNAs (siGLODErs) when introduced into human tumoral tissues [9]. Finally, we have also used the cationic polymeric particle INTERFERin^®^ (Polyplus-transfection SA) for transfecting antisense miRNA inhibitors into dopaminergic cells in culture, which provided neuroprotection against rotenone-induced cell damage [8]. Our experience indicates that choosing the proper nanoparticle is a critical step in preliminary planning and will affect the oligonucleotide efficacy directly.

Besides the significant technical advances of the aforementioned particles, developing carriers capable of delivery oligonucleotides in the brain is still an unmet need. Neurons are hard to transfect, and the blood–brain barrier represents an obstacle for delivering oligonucleotides into the central nervous system [9]. Magnetofection technology emerged as an effective strategy to overcome such limitations, allowing efficient delivery of functionally active oligonucleotides in vivo [46]. The present study tested the magnetic particle NeuroMag^®^ composed by polystyrene copolymers coated with iron, developed for transfecting primary neurons, as well as neuronal and glial cell lines. Soto-Sánchez et al. (2015) showed for the first time the capacity of this magnetoparticle to deliver nucleic acids in vivo [18]. NeuroMag^®^-complexed plasmids driven by a magnetic fields selectively transfected pyramidal cells of the rat visual cortex. Our data corroborated this finding but using a distinct genetic structure (a 9 Kb double-stranded circular plasmid DNA versus a short single-stranded oligonucleotide used here), as NeuroMag^®^ efficiently delivered Antimir-134 to cortical neurons, avoiding other cell types (Figure S1 of supplementary material). In both studies, the particle transfected nucleic acids into both the soma and apical dendrites of pyramidal neurons. Furthermore, the characterization data for the magnetic particles was consistent with our previous results [18]. The increase in average diameter of magnetic particles and decrease in superficial charge after conjugation with miRNA oligos indicate effective adsorption on the surface of Neuromag^®^. Changes in particle size may reflect the space filled by oligonucleotides around the particle while the reduction in zeta potential an electrical change may be caused by the negatively charged phosphate groups of nucleic acids [18,47].

Magnetofection with NeuroMag^®^ also presented an improved feature after i.c.v. injection. AntimiRs were efficiently biodistributed across the ependymal cell layer, reaching neurons and glial cells in the rat striatum. This finding highlighted the use of i.c.v. route for promoting a more widespread distribution of oligonucleotides in the striatum that is an anatomically extended brain structure in rostrocaudal direction. In summary, microRNAs inhibitors (AntimiRs) complexed with NeuroMag^®^ are capable of decreasing the content of targeted miRNAs overexpressed in brain diseases including PD, in which the striatal degeneration is a key pathological hallmark. 

In the present study, in addition to the magnetofection strategy used to enhance the oligonucleotides delivery, we also paid special attention to the AntimiR-134 chemical structure. We intended to cause silencing effects specifically on miR-134 and remaining for at least seven days after AntimiR-134 i.c.v. injection. These aims thus demanded a nucleotide molecule with increased specificity and stability. The oligos used our study were synthesized with two special modifications for improving nuclease stability with reduced immunogenicity and off-target effects: (i) change in the nucleotide ribose ring to make locked nucleic acids (LNA); and (ii) substitution of the phosphodiester linkages between nucleotides by phosphorothioate ones [9,40]. Our group has reported that the same AntimiR′s chemical changes employed in this study could produce an efficient down-regulation of a specific microRNA in dopaminergic cells in culture [8]. Therefore, the cell-specific miRNA silencing and the extended duration of knockdown are striking advantages for an RNAi-based therapeutics exploitable for chronic neurodegenerative diseases.

In conclusion, this work found that Neuromag^®^ is a valuable magnetic particle for delivering AntimiRs in vivo, and that magnetofection of i.c.v. injected oligos can suppress target miRNAs in striatum for at least 7 days. This RNAi-based therapeutic strategy could be of value for regulating pathological miRNAs overexpressed in the striatum, a brain area critically relevant for PD. The therapeutic potential of NeuroMag^®^-complexed miR-134 inhibitors in controlling underlying mechanisms of PD could be examined in further studies with pre-clinical models of PD.

## 4. Materials and Methods

### 4.1. Animals and Surgical Procedures

Female adults Sprague–Dawley rats (250–300 g) were used in the assays (*n* = 5 per group). The animals were housed in cages and kept in a room maintained at a controlled temperature of 23 °C and 55% humidity, with 12/12 h light-dark cycle. Throughout all the experiment period, food and water were available ad libitum. All experimental techniques approved by the Committee for Ethics in Animal Use of the University of Brasília, under UnBdoc No. 45524/2016.

### 4.2. Size and Zeta Potential Measurements

Particle size of magneto nanoparticle (Neuromag^®^, San Diego, CA, USA) and anti-miRNA magnetoplexes vectors was determined by dynamic light scattering (DLS) and their superficial charge by laser Doppler micro-electrophoresis. The measurements were taken on a Zetasizer Nano ZS (Malvern Instruments, Malvern, UK). Samples were diluted in NaCl 0.1 mM Milli-Q water) and all measurements were carried out in triplicate.

### 4.3. Surgical Procedures

We first carried out rat analgesia by using buprenorphine [0.025 mg kg^−1^ subcutaneous (s.c)]. Second, the anesthesia and sedation were induced by a cocktail of ketamine HCl (40 mg kg^−1^ i.p) and diazepam [5 mg kg^−1^ intraperitoneal (i.p)]. Anesthesia was maintained with a mix of oxygen and isofluorane (2%). During the procedure, the rats were monitored to: (a) depth of the anesthesia by continuous evaluation of the heart rate and blinking and toe pinch reflexes; (b) record of the O_2_ concentration levels in the blood; and (c) maintenance of the animals′ bodies warmed by using a thermal pad. 

For intracerebroventricular (i.c.v.) injections of NeuroMag^®^-complexed oligonucleotides this work used the following coordinates A: +0.7; L: −2.3; D: −5.2 from Bregma, according to the atlas of Paxinos and Watson (2015) [48]. We drilled a small craniotomy to expose the dura mater and arachnoids of the rat. Before the injection, fluorescein isothiocyanate (FITC)-labeled oligonucleotides (0.36 nmol in 0.36 μL; Exiqon A/S, Vedbaek, Denmark) were incubated with 0.68 μL of NeuroMag^®^ (Magnetofection™, OZ Biosciences USA Inc., San Diego, CA, USA) for at least 20 min at 37 °C. After that, a Hamilton 33-gauge needle micro-syringe (Hamilton Company, Reno, NV, USA) was held within the stereotaxic frame to inject in the right ventricle a total volume of 1 μL of the NeuroMag^®^-oligonucleotide solution for 10 min. The needle remained in situ for an additional 10 min before being removed slowly. A magnetic plate was placed underneath the head for 30 min to drive magnetic particles towards and into the brain cells, according to previous work [18] (Sóto-Sanches et al., 2015). After the surgical procedure, the animals were housed in their cages for 7 days, in a temperature and light-controlled room with a 12 h light/darkness cycle. A post-surgery treatment with antibiotics (enrofloxacin 25 mg kg^−1^ s.c.), anti-inflammatory and analgesic drugs (meloxicam 2 mg kg^−1^ s.c.) were administered. 

### 4.4. Tissue Processing

At Day 7 after the stereotaxic injection of Neuromag^®^-complexed oligos, rats were perfused under general anesthesia through the left ventricle of the heart with phosphate buffered saline (PBS; 10–15 min), followed by 4% paraformaldehyde (PFA; 10–15 min). Brains were removed and submitted to a 4% PFA for 48 h followed by sucrose gradient (10%, 20%, and 30%) in a shaker at room temperature. Next, the coronal sections of the rat brain were cut (20 μm) in a freezing microtome (CM1850, Leica, Wetzlar, Germany) throughout the rostrocaudal extent.

### 4.5. Immunohistochemistry of Nano-Structured Molecule in the Striatum

We first treated brain sections with 1 mM cupric sulfate (CuSO_4_) in 50 mM ammonium acetate buffer for removing autofluorescence. Then, sections were incubated for 1 h in 10% normal bovine serum (Jackson, West Grove, PA, USA) with 0.5% Triton X-100 for blocking unspecific staining. Next, the samples were incubated overnight at room temperature with chicken anti-EGFP (Invitrogen, 1:100, Carlsbad, CA, USA) and rabbit anti-NeuN (Millipore, 1:300, Darmstadt, Germany) antibodies diluted in PBS containing 0.5% Triton X-100. The sections were washed in PBS and incubated with secondary antibodies, donkey anti-rabbit IgG (Alexa Fluor 633-conjugated; 1:100; Invitrogen) and goat anti-chicken (IgG Alexa Fluor 555-conjugated; 1:100; Invitrogen, Carlsbad, CA, USA) for 1 h. This study employed Hoechst 33342 reagent for labeling cells nuclei. Finally, the brain sections were washed in PBS, mounted in fluoromount Vectashield (Vector Laboratories, Burlingame, CA, USA) and coverslipped for analysis by laser-confocal microscopy (Leica TCS SPE Microsystems GmbH, Wetzlar, Germany). Immunohistochemical controls were performed by omission of either the primary or secondary antibodies (data not shown). 

### 4.6. Quantification of Striatal miRNAs by Real-Time Quantitative Polymerase Chain Reaction (RT-qPCR)

The striatum tissue was manually dissected from euthanized animals at Day 7 post-injection of Neuromag^®^-complexed oligos, and frozen at −80 °C. miRNAs were isolated by mirVana™ miRNA Isolation Kit, and quantified by fluorometry (Qubit^®^ 2.0 firmware 3.11; Thermo Fisher Scientific, Inc., Waltham, MA, USA). Following the cDNA synthesis (TaqMan MicroRNA Reverse Transcription kit, Applied Biosystems; Thermo Fisher Scientific, Inc.), miR-134a was quantified by RT-qPCR using the TaqMan system (QuantStudio 12K Flex system, Applied Biosystems; Thermo Fisher Scientific, Inc.). The reaction mix contained 2 μL cDNA, 1 μL miRNA miR-134 specific primer or the internal control RNU6B (both from Thermo Fisher Scientific, Inc.), 10 μL TaqMan^®^ Fast Advanced Master Mix (Applied Biosystems; Thermo Fisher Scientific, Inc.) and milli-Q pure water to 20 μL. The qPCR program consisted of two initial cycles (50 °C for 2min, 95 °C for 20 s), with posterior 40 amplification cycles (95 °C for 1min, 60 °C for 1 min). Each run was executed in triplicate, and included negative RT (non-template) controls. Relative expression of microRNAs was determined by the delta delta CT method (2*^−ΔΔCt^*) [49]. 

### 4.7. Statistical Analysis 

Results were analyzed by using SPSS software (Statistical Package for Social Sciences 17.0; SPSS, Inc., Chicago, IL, USA). Student′s t-test was used to evaluate differences between paired groups. *P* < 0.05 was considered statistically significant.

## Supplementary Materials

The following is available online, Figure S1: Magnetofection of Neuromag^®^-complexed FITC-labeled oligos in cortical cells.
