# Supplementary material for: Delivery of miRNA-Targeted Oligonucleotides in the Rat Striatum by Magnetofection with Neuromag®
Source: Molecules. 2018 Jul 23;23(7):1825. doi: 10.3390/molecules23071825 (PMC6099620; doi:10.3390/molecules23071825)
Supplement: Supplementary file 1 [file molecules-23-01825-s001.pdf]

molecules

Supplementary Figures

Figure S1 – Magnetofection of Neuromag®-complexed FITC-labeled oligos in cortical cells.

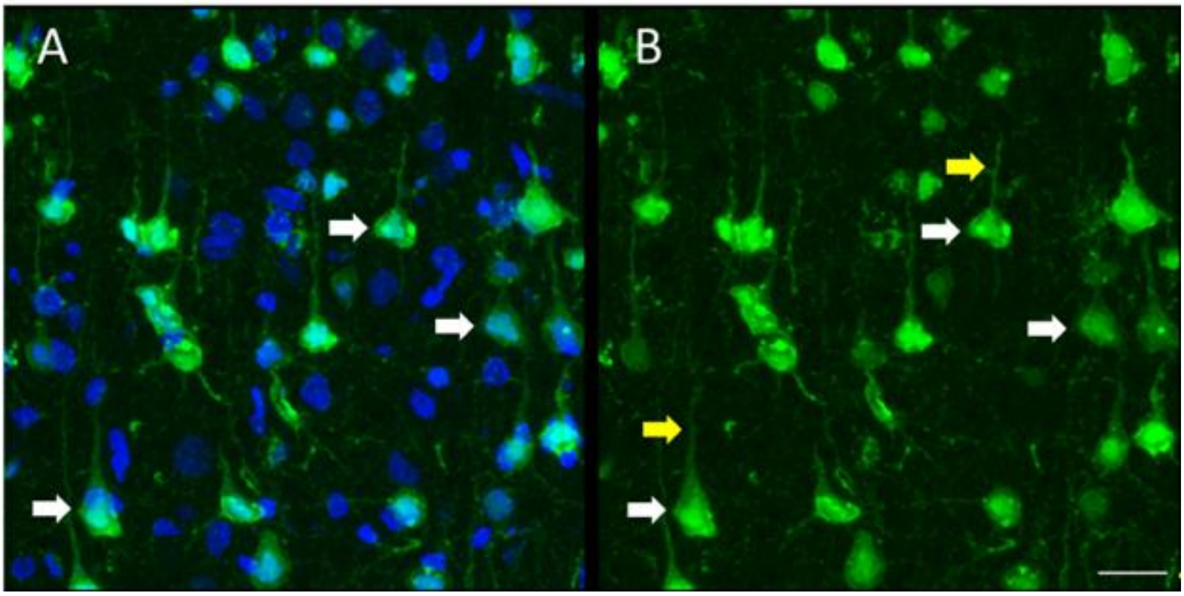

Figure S1. Fluorescence microscopy of brain cortical cells of rats injected with Neuromag®-complexed FITC-labeled oligonucleotides. The oligonucleotides were injected in rat cortex by stereotaxic surgery in animals anesthetized with isoflurane. In the right panel (**B**), a green fluorescence in soma (white arrows) and apical dendrites (yellow arrows) of pyramidal neurons of the brain cortex. On the left panel (**A**), the blue fluorescence of Hoescht 33342 nuclear staining in the same cells transfected with FITC-labeled oligos. Scale bar: 20 μm.
